# An Adaptive Fusion Attitude and Heading Measurement Method of MEMS/GNSS Based on Covariance Matching

**DOI:** 10.3390/mi13101787

**Published:** 2022-10-20

**Authors:** Wei Sun, Peilun Sun, Jiaji Wu

**Affiliations:** 1 College of Surveying, Mapping and Geographic Sciences, Liaoning Technical University, Fuxin 123000, China; 2 Research Center of Satellite Navigation and Positioning Technology, Wuhan University, Wuhan 430079, China

**Keywords:** multi-sensor fusion, combined attitude measurement, fusion filter, covariance matching, adaptive filter

## Abstract

Aimed at the problem of filter divergence caused by unknown noise statistical characteristics or variable noise characteristics in an MEMS/GNSS integrated navigation system in a dynamic environment, on the basis of revealing the parameter adjustment logic of covariance matching adaptive technology, a fusion adaptive filtering scheme combining innovation-based adaptive estimation (IAE) and the adaptive fading Kalman filter (AFKF) is proposed. By setting two system tuning parameters, for the process noise covariance adaptation loop and the measurement noise covariance adaptation loop, covariance matching is sped up and achieves an effective suppression of filter divergence. The vehicle-mounted experimental results show that the mean square error of the combined attitude error obtained based on the fusion filtering method proposed in this paper is better than 0.5°, and the mean square error of the heading error is better than 1.5°. The results can provide technical support for the continuous extraction of low-cost attitude information from mobile platforms.

## 1. Introduction

The development of multi-sensor data fusion technology is the key to realizing high-precision real-time attitude estimation [1,2,3]. Because of the nonlinearity of the heading and attitude system, its information fusion method is different from that of a linear system. At present, the common solutions to nonlinear problems are based on a mathematical model and described completely with conditional a posteriori probability, but actual nonlinear system operation is extremely difficult. The practical solution to the nonlinear problem is to linearize the mathematical model and approximate the sampling method, but these approaches have disadvantages, such as the linearization method having different degrees of truncation error, and the sampling approximation method having difficulty with guaranteeing numerical stability in principle [4,5,6]. A refined strong tracking unscented Kalman filter (RSTUKF) is proposed in refs [7]. This RSTUKF adopts the strategy of an assumption test to identify kinematic model errors and is developed to enhance UKF robustness against kinematic model errors. However, when the dimension is greater than 3, the statistical characteristics of the posterior distribution of some sigma points to nonlinear functions will be lost, and the estimation accuracy of the system will be reduced. Ref. [8] rigorously derives a novel adaptive CKF (Cubature Kalman filter) with fading memory for kinematic modelling errors and a new robust CKF with emerging memory for observation modelling errors, using the concept of Mahalanobis distance without involving artificial empiricism. However, CKF will discard part of the approximation error, which makes the filtering not meet the quasi consistency, so that it is unable to accurately estimate the true value of the state. The indirect Kalman filter is often used in the data processing of nonlinear systems because of its unique advantages, but it requires that the dynamic characteristics and noise statistical characteristics of the system be known. In the actual working environment, by setting two system tuning parameters for the process noise covariance adaptive loop and measurement noise covariance adaptive loop, the speed of covariance matching is accelerated, and the filter divergence is effectively suppressed. Affected by the measurement quality such as the multipath and internal measurement noise of the instrument, it is easy to cause the deterioration of the prior information setting, as well as a sudden change of the carrier itself, and the inaccurate setting of the initial value of the filter will affect the setting of prior information and lead to filter divergence. To solve this problem, one straightforward solution is to estimate the unknown noise statistics; Refs. [9,10] proposed the adaptive UKF method and the CKF method based on the maximum likelihood (ML) principle. On this basis, Ref. [11] proposes an adaptive UKF method combining the maximum likelihood principle and moving horizon estimation. On the other hand, Refs. [12,13] establish the random weighting estimations of system noise characteristics on the basis of the maximum a posteriori theory, and further develop a new Gaussian filtering method and an adaptive UKF method. On this basis, Ref. [14] combines the windowing and random weighting concepts and presents an adaptive UKF method. These methods, to a certain extent, weaken the filter divergence problem caused by unknown noise statistical characteristics.

There are also many adaptive filtering schemes. Theories and algorithms of random weighting estimation are established for estimating the covariance matrices of observation residual vectors and innovation vectors [15]. This can adaptively determine the covariance matrices of observation error and state error. Ref. [16] took into account the systematic errors of observation and kinematic models in the filtering process. This adaptively adjusts and updates the prior information through the equivalent weighting matrix and adaptive factor to resist the disturbances of systematic model errors on system state estimation, thus improving the accuracy of state parameter estimation. Ref. [17] presented a new adaptive square root unscented particle filtering algorithm by combining the adaptive filtering and square root filtering into the unscented particle filter. This can inhibit the disturbance of kinematic model noise and the instability of filtering data in the process of nonlinear filtering. Ref. [18] proposed an adaptive UKF with noise statistic estimator. This can estimate and adjust the system noise statistics online according to the covariance matching technology, which enhances the adaptive ability of standard UKF.

On the other hand, typical adaptive filtering schemes mainly include innovation-based adaptive filtering (innovation-based adaptive estimation, IAE) and fading adaptive Kalman filtering (the adaptive fading Kalman filter, AFKF) [19,20,21]. Because of the characteristics of adjusting the process noise covariance matrix and the measurement covariance matrix at the same time, the IAE method makes the corresponding covariance matrix show a nonpositive definite state in practical applications, resulting in filter divergence [22,23]. The traditional AFKF method has the limitation that the scale factor cannot be determined according to the dynamic environment and the accuracy of the observation model. Combined with the complementary characteristics of the two, an adaptive filtering method combining modified IAE and AFKF is proposed in this paper. By setting two system tuning parameters for the process noise covariance adaptive loop and the measurement noise covariance adaptive loop, the covariance matching speed is accelerated, and the filtering divergence is effectively suppressed.

## 2. MEMS/GNSS Combined Attitude

### 2.1. Error State Equation

Inertial navigation and satellite navigation have complementary advantages. Inertial navigation systems can provide rich navigation information and do not rely on external equipment, but they also have some shortcomings such as error divergence, and cannot provide time. Satellite navigation has the advantages of stable precision output and providing time references, but the signal is vulnerable to environmental interference and dynamic response lag [24,25,26]. Inertial/satellite tight integration can be used to estimate the attitude parameters of the carrier in a specific environment [27,28]. Assuming that the real state is $x_{t}$, the nominal state is x, and the error state is δx, the stochastic system model under the error state is deduced. The three are satisfied:

(1) $$x_{t}=x⊕\delta x$$

In the form, ⊕ stands for generalized addition. For the MEMS/GNSS system model, the real state space chooses quaternion $q_{b}^{n}$, velocity $v^{n}$, position $p^{n}$, gyroscope dynamic zero offset $b_{g}$ and accelerometer dynamic zero offset $b_{a}$. The nominal state determines the state vector of the Kalman filter:

(2) $$x_{t}={\left[\begin{array}{ccccc} {\left(q_{b}^{n}\right)}^{T} & {\left(v^{n}\right)}^{T} & {\left(p^{n}\right)}^{T} & {\left(b_{g}\right)}^{T} & {\left(b_{a}\right)}^{T} \end{array}\right]}^{T}$$

The differential equations of attitude quaternion, velocity and position are as follows:

(3) $$\{\begin{array}{c} {\dot{q}}_{b}^{n}=\frac{1}{2}\Omega (\omega (t))q_{b}^{n}\\ {\dot{v}}^{n}=a^{n}(t)\\ {\dot{p}}^{n}=v^{n}(t) \end{array}$$

In the form, $\omega (t)=[\begin{array}{ccc} \omega_{x} & \omega_{y} & \omega_{z} \end{array}{]}^{T}$ represents the output angle rate of the gyroscope in the ideal state, and $a^{n}$ indicates that the output acceleration in the ideal state is projected in the n-system.

There are still many random errors in the calibrated MEMS sensor. If the error is not estimated and compensated, the attitude error will accumulate until the solution is invalid. Therefore, the output of the MEMS accelerometer and gyroscope is modelled including random error factors:

(4) $$\begin{array}{c} \omega_{m}(t)=\omega (t)+b_{g}(t)+n_{g}(t)\\ a_{m}(t)=C(q_{b}^{n})(a_{m}(t)-g^{n})+b_{a}(t)+n_{a}(t) \end{array}$$

In the formula, $C(q_{b}^{n})$ represents the directional cosine matrix corresponding to the quaternion $q_{b}^{n}$; $g^{n}$ represents gravity vector; $n_{g}$ and $n_{a}$ represent the rate noise with zero mean Gaussian white noise characteristics; $b_{g}$ represents the dynamic zero offset of the gyroscope; and $b_{a}$ represents the dynamic zero offset of the accelerometer, which is driven by the Gaussian white noise vectors $n_{wg}$ and $n_{wa}$ with zero mean.

(5) $$\begin{array}{l} {\dot{b}}_{g}=n_{wg}\\ {\dot{b}}_{a}=n_{wa} \end{array}$$

The nominal state is obtained by combining Formula (3) and Formula (5):

(6) $$\{\begin{array}{c} {\dot{\hat{q}}}_{b}^{n}=\frac{1}{2}\Omega (\hat{\omega }(t)){\hat{q}}_{b}^{n}\\ {\dot{\hat{v}}}^{n}=C({\hat{q}}_{b}^{n}){\hat{a}}^{n}(t)+g^{n}\\ {\dot{\hat{p}}}^{n}={\hat{v}}^{n}(t)\\ {\dot{\hat{b}}}_{g}(t)=0_{3\times 1}\\ {\dot{\hat{b}}}_{a}(t)=0_{3\times 1} \end{array}$$

where:

(7) $$\begin{array}{l} {\hat{a}}^{n}(t)=a_{m}(t)-{\hat{b}}_{a}(t)\\ {\hat{\omega }}^{n}(t)=\omega_{m}(t)-{\hat{b}}_{g}(t) \end{array}$$

Generalized addition ⊕ is defined as the arithmetic addition rule between the nominal state and error state, which is used to deal with velocity error $\delta v^{n}$, positioning error $\delta p^{n}$, gyroscope dynamic zero bias error $\delta b_{g}$ and accelerometer dynamic zero bias error $\delta b_{a}$. For the quaternion error, the generalized error is explained by introducing error quaternion:

(8) $$\delta q=q_{t}⊗{\hat{q}}^{-1}\approx {\left[\begin{array}{cc} \frac{1}{2}\delta \theta^{T} & 1 \end{array}\right]}^{T}$$

The quaternion error δθ, velocity error $\delta v^{n}$, positioning error $\delta p^{n}$, gyroscope dynamic zero bias error $\delta b_{g}$ and accelerometer dynamic zero bias error $\delta b_{a}$ are selected to form a 15-dimensional error state vector:

(9) $$\delta x={\left[\begin{array}{ccccc} {\left(\delta \theta \right)}^{T} & {\left(\delta v^{n}\right)}^{T} & {\left(\delta p^{n}\right)}^{T} & {\left(\delta b_{g}\right)}^{T} & {\left(\delta b_{a}\right)}^{T} \end{array}\right]}^{T}$$

The error state model of a continuous stochastic system is obtained:

(10) $$\delta \dot{x}(t)=F\delta x(t)+GW$$

In the formula:

$$F=\left[\begin{array}{ccccc} -\left\lfloor \hat{\omega }\times \right\rfloor & 0_{4\times 3} & 0_{4\times 3} & -I_{4\times 3} & 0_{4\times 3}\\ -C_{b}^{n}\left\lfloor \hat{a}\times \right\rfloor & 0_{4\times 3} & 0_{4\times 3} & 0_{4\times 3} & C_{b}^{n}\\ 0_{4\times 3} & 0_{4\times 3} & I_{4\times 3} & 0_{4\times 3} & 0_{4\times 3}\\ 0_{4\times 3} & 0_{4\times 3} & 0_{4\times 3} & 0_{4\times 3} & 0_{4\times 3}\\ 0_{4\times 3} & 0_{4\times 3} & 0_{4\times 3} & 0_{4\times 3} & 0_{4\times 3} \end{array}\right]$$

$$G=\left[\begin{array}{cccc} -I_{4\times 3} & 0_{4\times 3} & 0_{4\times 3} & 0_{4\times 3}\\ 0_{4\times 3} & 0_{4\times 3} & C_{b}^{n} & 0_{4\times 3}\\ 0_{4\times 3} & 0_{4\times 3} & 0_{4\times 3} & 0_{4\times 3}\\ I_{4\times 3} & 0_{4\times 3} & 0_{4\times 3} & 0_{4\times 3}\\ 0_{4\times 3} & 0_{4\times 3} & I_{4\times 3} & 0_{4\times 3} \end{array}\right]$$

$$W=\left[\begin{array}{c} n_{g}^{}\\ n_{wg}^{}\\ n_{a}^{}\\ n_{wa}^{} \end{array}\right]$$

The continuous time noise covariance matrix *Q* and measurement noise covariance matrix *R* are calculated according to reference [29].

In practical applications, the Kalman stochastic model is generally derived in continuous time, and the continuous-time system needs to be equivalent to the corresponding discrete form. The deterministic system can be equivalently processed by Taylor expansion, and the stochastic system needs to test whether it still satisfies the basic noise hypothesis of the Kalman filter after equivalent processing. Taking the sampling interval of the MEMS gyroscope as the discretization interval $T_{s}=t_{k}-t_{k-1}$, the approximate equivalent discretization process of a continuous stochastic system is obtained:

(11) $$X_{k}=\Phi_{k/k-1}X_{k-1}+\Gamma_{k-1}W_{k-1}$$

In the formula:

(12) $$X_{k}=X(t_{k})$$

(13) $$\begin{array}{ll} \Phi_{k/k-1} & {=e}^{F(t_{k-1})T_{s}}\\ e^{F(t_{k-1})T_{s}} & =I+F(t_{k-1})T_{s}\\ & +F^{2}(t_{k-1})\frac{T_{s}^{2}}{2!}\\ & +F^{3}(t_{k-1})\frac{T_{s}^{3}}{3!}+\ldots \\ & \approx I+F(t_{k-1})T_{s} \end{array}$$

(14) $$\Gamma_{k-1}\approx \left[I+\frac{1}{2}F(t_{k-1})T_{s}\right]G(t_{k-1})\approx G(t_{k-1})$$

The discretization process of the system noise covariance matrix is as follows (15):

(15) $$Q_{d}=\int_{t_{k}}^{t_{k}+T_{s}}\Phi (t_{k+1},\tau )GQG^{T}\Phi (t_{k+1},\tau )d\tau$$

The discretization process of the one-step prediction mean square error matrix is as follows:

(16) $$P_{k/k-1}=\Phi_{k-1}P_{k}\Phi_{k-1}^{T}+Q_{d}$$

### 2.2. Measurement Equation

The measurement equation is in discrete form in most environments. Averaging the measurement equation within the discretization interval is the essence of the discretization continuous measurement equation. In the MEMS/GNSS integrated attitude estimation scheme, select the difference between the velocity information ${\hat{v}}_{ins}^{n}$ and position information ${\hat{p}}_{ins}^{n}$ calculated by the inertial navigation system, and the velocity information ${\hat{v}}_{gnss}^{n}$ position information ${\hat{p}}_{gnss}^{n}$ calculated by the satellite navigation system:

(17) $$Z_{k}=\left[\begin{array}{c} {\hat{v}}_{ins}^{n}-{\hat{v}}_{gnss}^{n}\\ {\hat{p}}_{ins}^{n}-{\hat{p}}_{gnss}^{n} \end{array}\right]=H_{k}\delta x_{k}+V_{k}$$

In the formula, V represents the noise measurement vector and the measurement matrix $H_{k}$ uses the chain rule to calculate the partial derivative:

(18) $$H_{k}≜{\frac{\partial H}{\partial \tilde{x}}|}_{x}={\frac{\partial H}{\partial x}|}_{x}{\frac{\partial x}{\partial \tilde{x}}|}_{x}=H_{x}X_{\tilde{x}}$$

In the formula, partial $H_{x}=\left[\begin{array}{ccc} 0_{6\times 3} & I_{6\times 6} & 0_{6\times 6} \end{array}\right]$ represents the partial derivative of the measurement information to the whole state, and $X_{\tilde{x}}$ represents the partial derivative of the state vector to the error state vector:

(19) $$X_{\tilde{x}}≜{\frac{\partial x}{\partial \tilde{x}}|}_{x}=\left[\begin{array}{ccc} \Theta & 0_{4\times 6} & 0_{4\times 6}\\ 0_{6\times 3} & I_{6\times 6} & 0_{6\times 6}\\ 0_{6\times 3} & 0_{6\times 6} & I_{6\times 6} \end{array}\right]$$

In the formula:

(20) $$\Theta =\frac{\partial q⊗\delta q}{\partial \theta }=\frac{1}{2}\left[\begin{array}{ccc} -q_{2} & -q_{3} & -q_{4}\\ q_{1} & -q_{4} & q_{3}\\ q_{4} & q_{1} & -q_{2}\\ -q_{3} & q_{2} & q_{1} \end{array}\right]$$

### 2.3. Filter Reset

According to standard Kalman filtering theory, the estimated value of the error state vector is iteratively updated. After the indirect Kalman filter is updated, the error state is combined with the nominal state by using the generalized addition combination method described in Formula (1), and the estimated value of the system state vector is obtained. After the error state is injected into the nominal state, the error state and its covariance matrix will be reset:

(21) $$\begin{array}{l} \delta \hat{x}\leftarrow 0\\ P\leftarrow MPM^{T} \end{array}$$

In the formula:

(22) $$M=\left[\begin{array}{cc} I_{3}+\left\lfloor \frac{1}{2}\delta \hat{\theta }\times \right\rfloor & 0_{3\times 12}\\ 0_{12\times 3} & I_{12\times 12} \end{array}\right]$$

Based on Equation (22), more accurate results can be produced, but indirect Kalman filtering is used in most cases, which usually simplifies the reset mode, that is, M = I.

## 3. Parameter Adaptive Logic Adjustment

In the case of accurate modelling, the covariance matrix of innovation theory should be approximately equal to the covariance of its statistical samples. In the case of inaccurate modelling, the innovation variance mismatch is caused by the deviation of noise parameters $Q_{k}$ and $R_{k}$. When the trace of the variance of the innovation statistical sample is much larger than that of the covariance matrix of the innovation theory, a serious innovation mismatch is generally considered to be caused by the gross error of the measurement information, so the measurement should be isolated in time without updating the Kalman filter measurement.

When the innovation mismatch is not serious, the covariance matching technique which introduces the proportional coefficient to adjust the noise parameters $Q_{k}$ and $R_{k}$ can be used. When the covariance of the innovation statistics sample is near the theoretical innovation covariance, it shows that the two covariances almost match. If the covariance of innovation statistics is greater than its theoretical value, the noise parameter value needs to be reduced, and if the actual covariance is less than its theoretical value, the noise parameter value should be increased. The update method is as follows:

(23) $$\begin{array}{cc} Q_{k}=\alpha Q_{k} & R_{k}=\beta R_{k} \end{array}$$

(24) $$\begin{array}{cc} Q_{k}=\alpha^{-(k+1)}Q_{k} & R_{k}=\beta^{-(k+1)}R_{k} \end{array}$$

In the formula, α and β are the adjustment scale coefficients. Taking Equation (23) as an example, the gain matrix $K_{k}^{}$ of the adaptive Kalman filter can be changed to:

(25) $$K_{k}^{}=P_{k/k-1}H_{k}^{T}{\left(H_{k}P_{k/k-1}H_{k}^{T}+\beta R_{k}\right)}^{-1}$$

(26) $$P_{k/k-1}=\Phi_{k/k-1}P_{k-1}\Phi_{k/k-1}^{T}+\Gamma_{k-1}(\alpha Q_{k-1})\Gamma_{k-1}^{T}$$

To measure the degree of innovation mismatch, the degree of mismatch (degree of mismatch, DOM) is used to describe:

(27) $$DOM=\frac{tr({\hat{C}}_{v_{k}})}{tr(C_{v_{k}})}$$

${\hat{C}}_{v_{k}}$ represents the covariance of innovation statistics and $C_{v_{k}}$ represents the covariance of theoretical innovation. When the DOM is approximately equal to the set threshold, that is, the innovation statistical covariance based on the sampling sequence is approximately equal to the theoretical innovation covariance, the noise parameters should remain unchanged. When the DOM is larger than the set threshold, that is, the trace of the innovation statistical covariance is larger than that of the theoretical innovation covariance, it is necessary to increase the process noise statistics. When the DOM ratio is small, that is, the trace of the innovation statistical covariance is smaller than that of the theoretical innovation covariance, it is necessary to reduce the process noise statistics.

## 4. Adaptive Kalman Filtering Method Combining Innovation and Fading

By calculating the maximum likelihood estimation of the innovation variance, the IAE method modifies the Kalman filtering gain directly from the actual innovation calculation to improve the Kalman filtering robustness when the satellite navigation system measurement changes greatly. By introducing the suboptimal scaling factor, the AFKF can reduce the inertia of the filter by changing the ratio of system noise and measurement noise to improve the tracking ability in a dynamic environment. By combining the complementary characteristics of the two, a new adaptive scheme is proposed in this paper. In the scheme, two system tuning parameters, the forgetting factor and the noise covariance scaling factor, are provided. When the filter reaches the optimal estimation, the actual innovation covariance and the theoretical innovation covariance based on the sampling sequence should be equal. This method has the advantages of high computational efficiency and good numerical stability, and can avoid losing the positive definiteness of the matrix in the Kalman filtering cycle. According to the theoretical covariance matrix of the innovation sequence, an adaptive scheme based on the covariance matching technique is obtained by setting two system tuning parameters: the forgetting factor and the noise covariance scaling factor.

(28) $$C_{v_{k}}^{*}=H_{k}(\lambda_{p}P_{k/k-1})H_{k}^{T}+\lambda_{R}R_{k}$$

The change in covariance is basically determined by two parameters:

(29) $$\{\begin{array}{c} P_{k/k-1}^{*}=\lambda_{p}P_{k/k-1}\\ R_{k}^{*}=\lambda_{R}R_{k} \end{array}$$

In the formula, $\lambda_{p}$ and $\lambda_{R}$ denote the forgetting factor and noise covariance scaling factor, respectively. Moreover, considering that $\lambda_{p}$ should not be less than 1, the calculation process is obtained:

(30) $$\{\begin{array}{c} \begin{array}{cc} \left(\lambda_{p}{)}_{ii}=\max(\begin{array}{cc} 1 & \frac{tr({\hat{C}}_{v_{k}})}{tr(C_{v_{k}})} \end{array}\right) & i=1,2,\ldots ,m \end{array}\\ \begin{array}{cc} (\lambda_{R}{)}_{jj}=\frac{tr({\hat{C}}_{v_{k}})}{tr(C_{v_{k}})} & i=1,2,\ldots ,n \end{array} \end{array}$$

Although both $\lambda_{p}$ and $\lambda_{R}$ are related to the covariance of innovation statistics, the moving windows are $N_{p}$ and $N_{R}$, respectively. Different filtering methods have different factor settings. Reference [30] gives a specific description of the value. When performing the process noise covariance adaptation, it is necessary to obtain the latest information of the measurement noise intensity in advance; otherwise, innovation covariance matrix deviation will occur due to the inability to detect the measurement noise correctly, resulting in unreliable results. This problem can be avoided by using two different window sizes: one for the process noise covariance adaptive loop and the other for measuring the noise covariance adaptive loop.

According to the basic formula of the Kalman filter, the covariance matrix of the measurement updating phase is obtained.

(31) $$P_{k}^{*}=(I-K_{k}^{*}H_{k})P_{k/k-1}^{*}$$

The gain $K_{k}^{*}$ of the improved adaptive Kalman filter can be expressed as:

(32) $$K_{k}^{*}=P_{k/k-1}^{*}H_{k}^{T}{\left(H_{k}P_{k/k-1}^{*}H_{k}^{T}+R_{k}^{*}\right)}^{-1}$$

## 5. Experimental Results and Analysis

**Figure 1:**
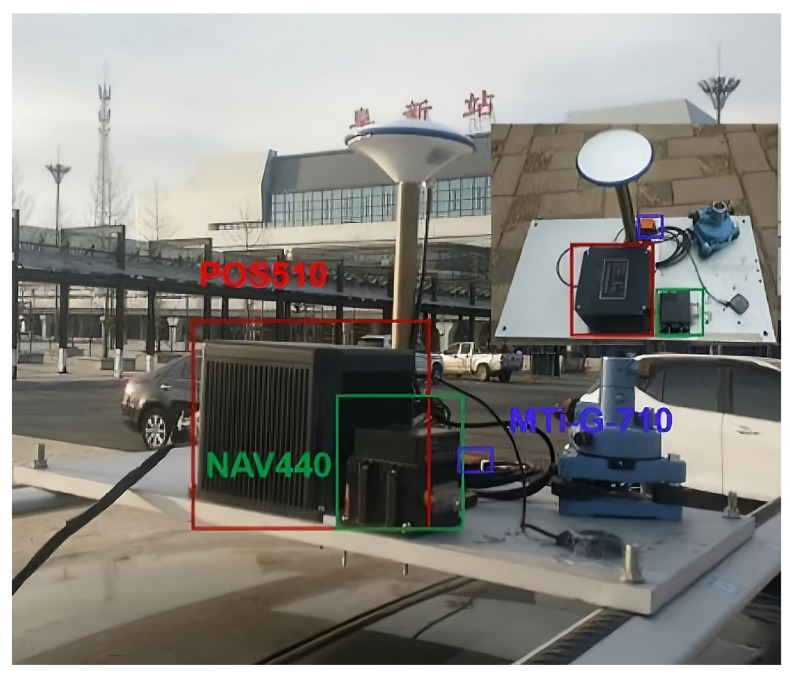
Construction of experimental environment.

The test platform is shown in Figure 1, the inertial sensor and the satellite antenna are fixed on the customized experimental platform, and the relative position relationship between the sensors is known. The combination result of POS510 and GPS RTK is used as the reference. The installation information of the rod arm is 0.022 quill 0.141 meme 0.282 (single bit: m). Low-cost inertial data are collected via MTi-G and NAV440 at a 100 Hz sampling rate. Time alignment is carried out between systems through GPS.

### 5.1. Verification Experiment of Algorithm Feasibility

**Figure 2:**
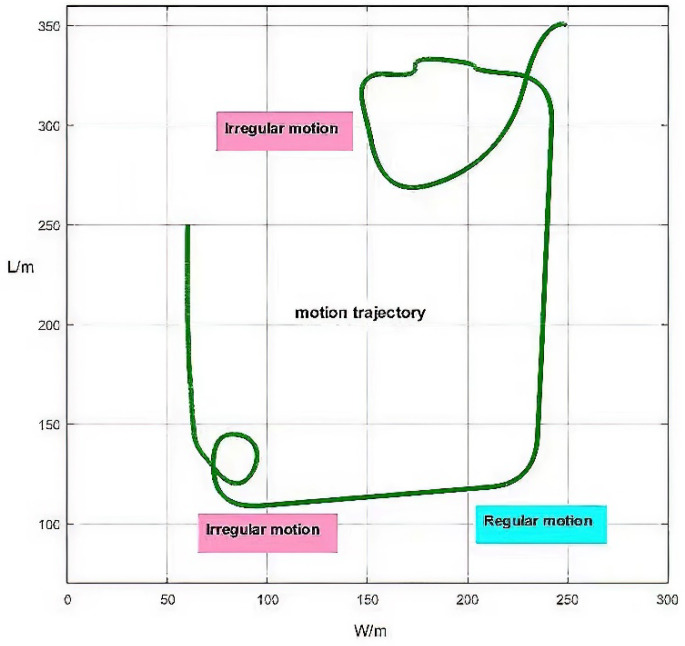
Motion trajectory diagram.

The data involved in this experiment are provided by MTi-G. The sampling frequency of the MTi-G inertial measurement unit is set to 100 Hz. The experiment was carried out on a sports field with a length of 400 m and a width of 300 m. The sensor was placed on a trolley. At first, it moved irregularly, then it moved regularly along a straight line, and finally it moved irregularly again. The motion trajectory is shown in Figure 2. The MTi-G performance parameters are shown in Table 1.

The 15-dimensional error vector is used as the state quantity of Kalman filtering, and the relevant parameters are set as follows: the initial velocity error is 0.01 m/s, the initial position error is 0.01m, the acceleration zero offset initial value is 0.3 m/s^2^, the gyro zero bias initial value is 0.3°/s, the accelerometer noise is *σ_a_* = 0.5 m/s^2^, the gyro noise is *σ_g_* = 1°/s, the accelerometer zero bias drive noise is *σ_ba_* = 10^−5^ m/s^2^, and the gyro zero bias drive noise is *σ_bg_* = 10^−5^°/s.

Around the four data processing methods set below, the low dynamic attitude experiments are carried out using the data collected by the MEMS/GNSS integrated system, and the corresponding attitude estimation results are obtained for comparison.

Experiment 1: General NoBaro filter provided by Xsens Company.

Experiment 2: IAE Kalman filtering algorithm, innovation adaptive moving window N is set to 20.

Experiment 3: AFKF filtering algorithm.

Experiment 4: The adaptive scheme of combining IAE and AFKF designed in this paper, the mobile windows $N_{p}$ and $N_{R}$ are set to 20 and 30, respectively.

The results obtained by General NoBaro filtering are used as a reference to test the relative accuracy from Experiment 2 to Experiment 4.

**Figure 3:**
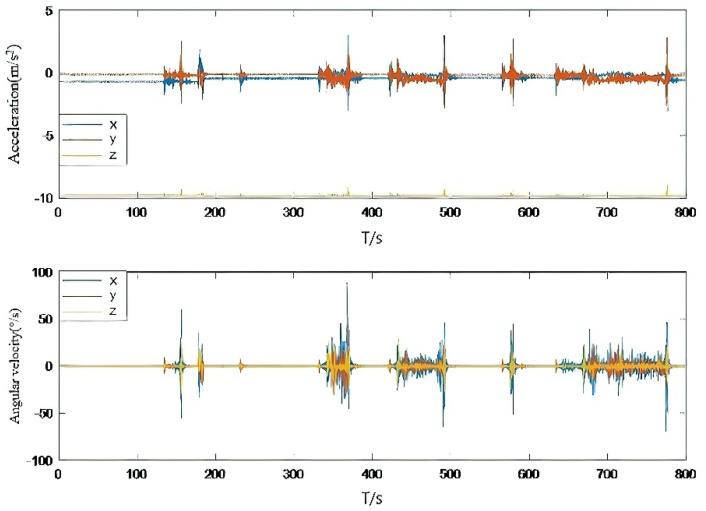
Accelerometer data and gyroscope data.

**Figure 4:**
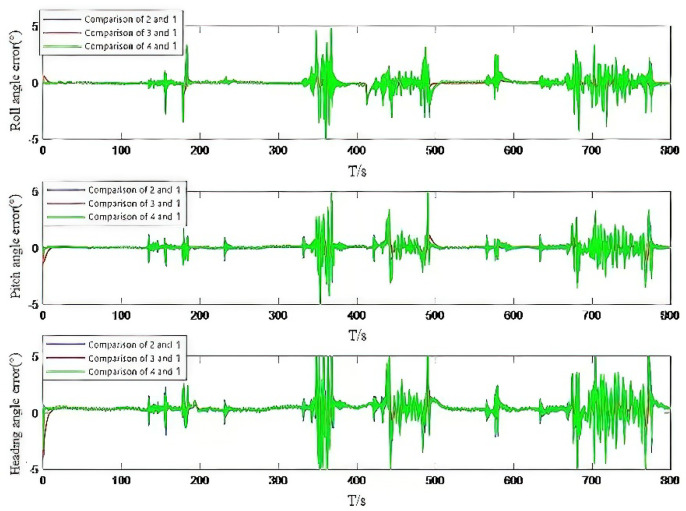
Attitude angle error of measured data.

Combined with the analysis of Figure 3 and Figure 4, we can see that when the accelerometer and gyroscope data change, although Experiment 2 and 3 can slow down the degree of innovation variance mismatch to some extent, the mitigation is not enough. In this paper, a fusion adaptive scheme of IAE and AFKF is proposed, which can quickly achieve covariance matching by setting two tuning factors, and the solution result is obviously better than that of Experiment 2 and Experiment 3. Table 2 shows the statistical values of the mean square error, maximum error, minimum error and average error of attitude angle error, obtained by comparing the IAE method, AFKF method, fusion IAE and AFKF adaptive method proposed in this paper with the reference data solution results.

Through the analysis of Table 2, it can be seen that the mean square error of the attitude angle is less than 0.5°, the average error is less than 0.2°, the mean square error of the heading angle is less than 1.5°, and the average error is less than 1°. It is verified that the proposed algorithm can provide scheme support for the continuous acquisition of MEMS/GNSS attitude system parameters.

### 5.2. City Car-Borne Experiment in Urban Environment Experiment

**Figure 5:**
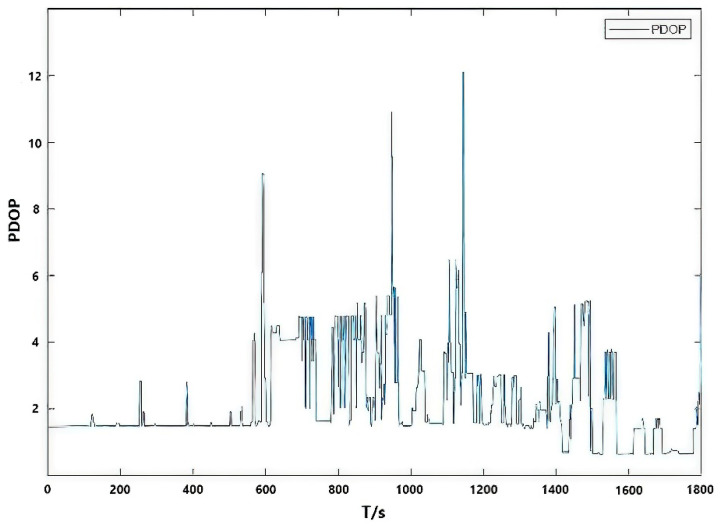
PDOP value in the urban experimental environment.

**Figure 6:**
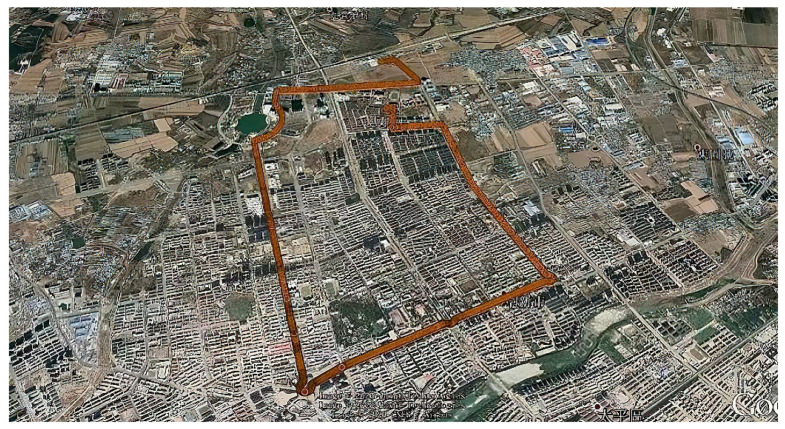
Test track diagram.

On board test data will be collected on 7 June 2021. The model of car used in the experiment is a Skoda Rapid Spaceback. The choice of driving path includes three characteristics: open sky, urban block and urban canyon. The total length of the route is 13 km. The vehicle is stationary for the first ten minutes. After starting, the average driving speed is 50 km/h. In the complex urban environment, the satellite signal will be interfered to varying degrees, and the satellite signal occlusion in the urban canyon environment is so serious that it cannot even receive the satellite signal, which can be seen from the PDOP value of Figure 5. The difference in measurement information quality can better verify the performance of the fusion filtering algorithm proposed in this paper, based on the above complex environment analysis combined with IAE and AFKF adaptive indirect Kalman filtering scheme performance. The trajectory of the carrier is shown in Figure 6.

The satellite navigation system provides the initial position and initial velocity of the moving carrier during the experiment, and the inertial navigation system provides the initial attitude information. For high precision inertial navigation, the horizontal alignment in the initial alignment process can be completed quickly, and the azimuth alignment process takes 5–30 min to complete. For an MEMS inertial navigation system, the initial alignment process needs to be assisted by a satellite navigation system.

**Figure 7:**
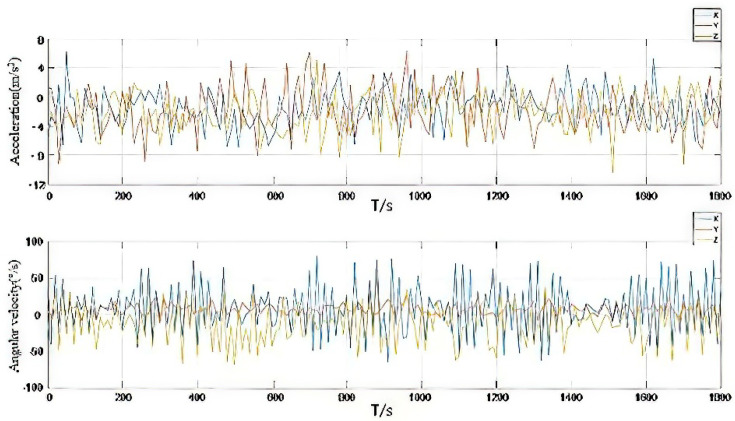
Accelerometer data and gyroscope data.

**Figure 8:**
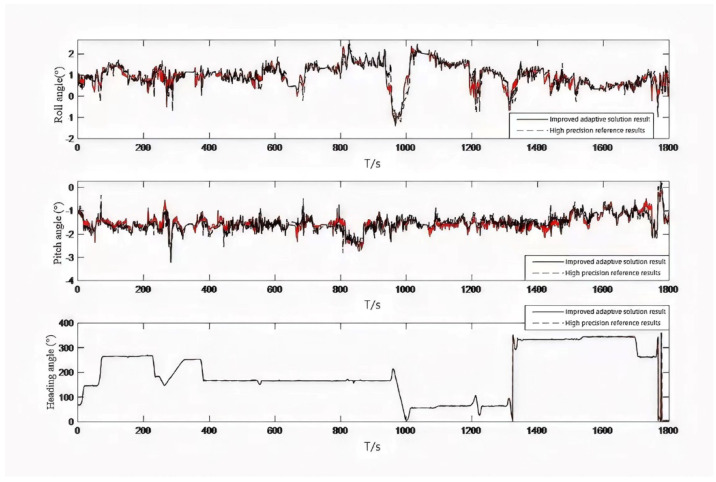
Comparison curve of attitude angle solution.

**Figure 9:**
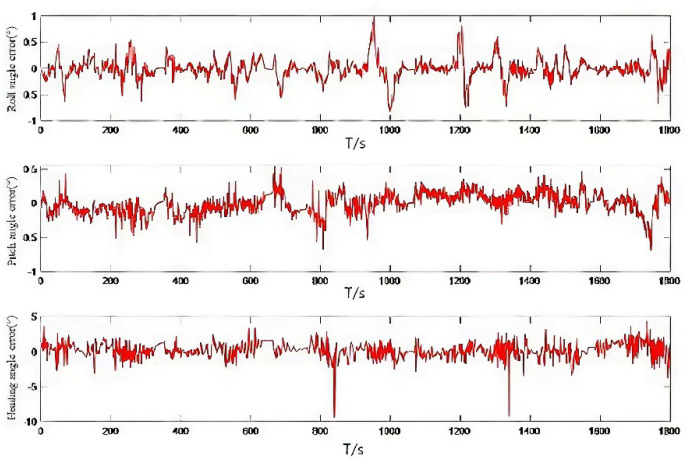
Attitude angle error.

Accelerometer data and gyroscope data are shown in Figure 7. The attitude curve obtained by the fusion filtering method proposed in this paper is shown in Figure 8. As seen from the figure, in the complex program environment, the algorithm proposed in this paper is still consistent with the high-precision reference change, to verify the absolute reliability of the algorithm. The attitude error map is shown in Figure 9. By calculating its statistical characteristics, it is found that the mean square error of the roll angle is 0.3018°, the mean square error of the pitch angle is 0.4756°, and the mean square error of the heading angle is 1.4218°. The comparison with the high-precision reference system shows that the mean square error of attitude error is less than 0.5° and the mean square error of heading angle is less than 1.5°. Its absolute accuracy can provide technical support for the continuous extraction of low-cost inertial/satellite heading attitude parameters.

It is noteworthy that the variations in noise estimation are not presented here because both experiments were in dynamic scenarios; the stimulated dynamical noises contain not only the filter estimation errors, but also the outliers induced by the complex experimental conditions. This was especially significant for the car-driving test in the urban environment, where the road conditions affected the test data significantly. Therefore, the variations in the noise estimations, i.e., the covariance components of the estimated attitude angles, did not show an apparent convergency trend, as one expected in some low dynamic experiments.

## 6. Conclusions

This paper reveals the parameter adjustment logic of covariance matching adaptive technology. Based on the existing problems of the IAE and AFKF methods, a new adaptive filtering scheme combining the two methods is proposed. Two system tuning parameters are set for the adaptive loop of process noise covariance and the adaptive loop of measuring noise covariance to accelerate the speed of covariance matching. Vehicle experiments in low dynamic open ground and high dynamic urban environments verify the performance of the proposed algorithm in different scenarios. The experimental results show that the mean square error of the horizontal attitude error is better than 0.5° and the mean square error of the heading error is better than 1.5°. The results can provide technical support for the continuous acquisition of dynamic parameters.

## Figures and Tables

**Table 1:** Performance parameters of the inertial sensor.

	Gyroscope	Accelerometer
Full range	±1000°/s	±18 g
Bias stability	10 °/h	40 μg
Noise density	0.01°/s/√Hz	80 μg/√Hz
Nonlinearity	0.01 %	0.03 %
Full range	±1000°/s	±18 g

**Table 2:** Attitude angle calculation error.

**a.** Attitude angle calculation error of IAE method			
	**Roll Angle**	**Pitch Angle**	**Heading Angle**
Mean square error	0.3180°	−0.5892°	−1.3980°
Maximum error	3.7212°	2.7815°	8.5192°
Minimum error	−4.2544°	−2.7833°	−7.4890°
Average error	−0.2036°	0.3023°	0.8874°
**b.** Attitude angle calculation error of AFKF method			
	**Roll angle**	**Pitch angle**	**Heading angle**
Mean square error	0. 4710°	0.2750°	1.3212°
Maximum error	2.9892°	2.8896°	8.0192°
Minimum error	−3.9210°	−3.1158°	−7.8504°
Average error	0.1138°	0.1484°	0.8249°
**c.** Attitude angle calculation error of fusion IAE and AFKF adaptive method			
	**Roll angle**	**Pitch angle**	**Heading angle**
Mean square error	0.1180°	0.2471°	1.2633°
Maximum error	1.9952°	2.3727°	4.8672°
Minimum error	−3.7170°	−2.7108°	−7.4280°
Average error	−0.0037°	0.1136°	0.8029°

## Data Availability

Not applicable.
